# SARS-CoV-2 and diabetes: a post-pandemic reappraisal

**DOI:** 10.1007/s00125-026-06785-4

**Published:** 2026-06-27

**Authors:** Noora Said, Ian Jones, Emily K. Anderson-Baucum, Carmella Evans-Molina

**Affiliations:** 1 Department of Pediatrics, Indiana University School of Medicine, Indianapolis, IN USA; 2 Center for Diabetes and Metabolic Diseases, Indiana University School of Medicine, Indianapolis, IN USA; 3 Herman B Wells Center for Pediatric Research, Indiana University School of Medicine, Indianapolis, IN USA; 4 Department of Medicine, Indiana University School of Medicine, Indianapolis, IN USA; 5 Richard L. Roudebush VA Medical Center, Indianapolis, IN USA

**Keywords:** Beta cell, COVID-19, Diabetes mellitus, Long COVID, Pancreas, Review, SARS-CoV-2

## Abstract

The COVID-19 pandemic was a dynamic and often confusing period for clinical and biomedical research. As severe acute respiratory syndrome coronavirus 2 (SARS-CoV-2) spread globally, knowledge accumulated rapidly through publications that were frequently based on preliminary or sometimes conflicting evidence, yet these papers played a critical role in shaping evolving medical, research and societal responses. Early in the pandemic, diabetes emerged as one of the strongest predictors of severe COVID-19 outcomes and mortality, placing it at the centre of early risk-stratification and therapeutic frameworks and prompting urgent efforts to understand the biological basis of these associations. As the pandemic progressed, reports of new-onset diabetes following COVID-19 infection raised the possibility of a bidirectional relationship between SARS-CoV-2 infection and diabetes. In this review, we provide a post-pandemic reappraisal of the clinical and experimental literature examining the intersection of COVID-19 and diabetes. We summarise proposed pathophysiological mechanisms, including the effects of SARS-CoV-2 infection in the pancreas and on peripheral insulin-sensitive tissues. We review key meta-analyses assessing the association between COVID-19 and incident type 1 and type 2 diabetes and highlight strengths and weaknesses of the epidemiologic studies underpinning these findings. We highlight the highest-quality evidence from prospective cohorts, as well as relevant clinical trials and registry-based studies that emerged from this collective experience. We discuss emerging relationships between long COVID and diabetes and the effect of vaccination on diabetes risk following SARS-CoV-2 infection. Finally, we identify critical knowledge gaps and outline priorities for ongoing and future studies needed to resolve remaining uncertainties.

**Graphical Abstract:**

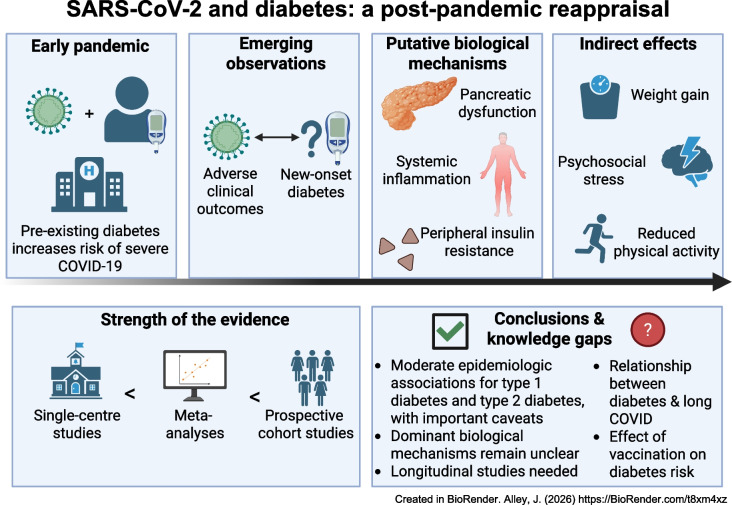

**Supplementary Information:**

The online version contains a slide of the figure for download available at 10.1007/s00125-026-06785-4.

## Introduction

In the autumn of 2019, reports of a mysterious new respiratory virus emerged from China, marking the beginning of a global health crisis that would eventually touch all corners of the world. As severe acute respiratory syndrome coronavirus 2 (SARS-CoV-2) spread, clinicians and policy makers were forced to rapidly assimilate fragmentary data, adapt to shifting treatment paradigms and make critical decisions in real time, often based on preliminary or conflicting evidence. Although SARS-CoV-2 was initially characterised as a respiratory pathogen, clinical experience soon revealed it to be a systemic and multi-organ disorder. In addition to the lung parenchyma and respiratory epithelia [1], SARS-CoV-2 was shown to infect and cause acute and chronic sequelae in diverse tissues, including the cardiac myocardium [2], renal tubular epithelium [3], intestinal cells [4] and vascular tissue [5].

At the same time, it became clear that certain groups of individuals experienced disproportionately severe outcomes, including higher rates of hospitalisation, intensive care use and mortality. Early epidemiological studies identified several demographic factors associated with poor outcomes, most notably older age, male sex, obesity and lower socioeconomic status [6, 7]. In addition, data from early clinical cohorts rapidly established that a pre-existing diagnosis of diabetes was one of the most potent predictors of adverse COVID-19 outcomes. In initial hospitalised populations from Wuhan, China, diabetes was significantly over-represented among non-survivors [8], foreshadowing the elevated mortality later confirmed in larger datasets across multiple continents.

Population-scale analyses from England, including the OpenSAFELY and national diabetes registries, demonstrated that both type 1 and type 2 diabetes were independently associated with markedly higher COVID-19-related mortality, even after adjustment for demographic and clinical features [7, 9]. Subsequent studies in critically ill cohorts, United States insurance claims databases and health networks reinforced these observations, with higher haemoglobin A_1c_ in those with existing diabetes linked to progressively worse outcomes [10–12]. Early data further suggested that in acute COVID-19, the prevalence of diabetic ketoacidosis and acute hyperglycaemic crises amongst individuals with pre-existing diabetes was higher [13], while rates of diabetic ketoacidosis in children and adolescents presenting with new-onset type 1 diabetes were also increased [14–16]. Soon, additional studies began to identify similar increases in new-onset type 2 diabetes [17].

These clinical observations placed diabetes squarely at the centre of early risk-stratification frameworks, influencing social distancing recommendations, vaccination prioritisation and therapeutic management strategies [18]. They also prompted urgent efforts to understand why SARS-CoV-2 and diabetes intersected so powerfully and raised important questions regarding the nature of this relationship. While individuals with established diabetes were clearly at increased risk for severe COVID-19 outcomes, accumulating reports also described hyperglycaemia and cases of new-onset diabetes following infection [7–10, 14–16, 19, 20]. Determining whether and how SARS-CoV-2 infection might influence diabetes development became an area of intense investigation during the pandemic.

**Fig. 1:**
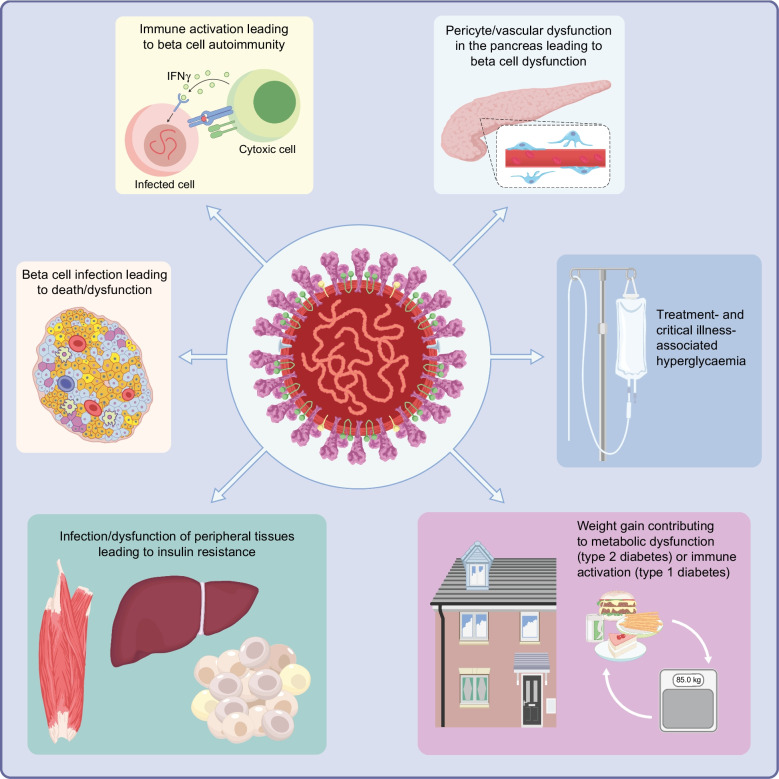
SARS-CoV-2 is associated with pleiotropic effects in the pancreas, insulin-sensitive tissues and immune system, while COVID-19 treatment and containment strategies may modify glucose homeostasis, psychosocial stress levels and diabetes development. This figure is available as a downloadable slide

In this review, we provide a post-pandemic reappraisal of the evidence linking SARS-CoV-2 infection and diabetes. Synthesis of these data suggests that SARS-CoV-2 predisposes to altered glucose homeostasis through a variety of pathways, including direct effects on the pancreas and insulin-sensitive tissues and indirect effects on systemic inflammation and lockdown-associated changes in diet, weight, physical activity and psychosocial stress (Fig. 1). Here, we summarise these proposed pathophysiologic mechanisms, integrating data across pancreatic, peripheral and systemic pathways, through which the virus and host inflammatory responses may influence both type 1 and type 2 diabetes risk and pathogenesis. We then examine epidemiologic data evaluating the relationship between COVID-19 and incident diabetes, and we highlight both supportive findings and key methodological limitations that complicate causal inference. Finally, we discuss insights emerging from prospective cohorts and long-term follow-up studies, and we identify critical knowledge gaps that remain as the metabolic consequences of SARS-CoV-2 infection continue to evolve.

This narrative review was informed by targeted PubMed searches using combinations of the terms ‘COVID-19’, ‘SARS-CoV-2’ and ‘diabetes’, including both ‘type 1 diabetes’ and ‘type 2 diabetes’, as well as ‘beta cells’, ‘peripheral insulin signalling’, ‘long COVID’ and ‘autoantibody-positive’. Our searches encompassed the period from the onset of the pandemic to the present. Studies were selected based on relevance to proposed mechanisms, clinical observations and epidemiologic associations linking SARS-CoV-2 infection with dysglycaemia and diabetes. Where available, we incorporated findings from systematic reviews and meta-analyses, considered the strengths and limitations of these approaches and reviewed reference lists of articles to identify additional relevant studies.

## Pathophysiologic links between SARS-CoV-2 infection and diabetes

### Direct effects of SARS-CoV-2 on the pancreas

Early clinical observations of increased rates of diabetic ketoacidosis at presentation in individuals with newly diagnosed diabetes prompted investigation into potential effects of SARS-CoV-2 on pancreatic beta cell function. The publication of a case report in October 2020 describing autoantibody-negative, insulin-dependent diabetes in a young male individual following COVID-19 infection [21] intensified speculation of direct viral effects on the pancreas. Although a single case study could not establish causality, several months earlier in July 2020, Yang and colleagues had reported that human pluripotent stem cell (hPSC)-derived pancreatic cells and adult primary human islets were permissive to SARS-CoV-2 infection [22]. What followed was a rapid succession of papers that painted an interesting but confusing picture regarding the cell-type specificity and physiological relevance of SARS-CoV-2 infection within the pancreas. Multiple studies published in late 2020 to mid 2021 reported the presence of the SARS-CoV-2 entry factors, including angiotensin-converting enzyme 2 (ACE2) and transmembrane serine protease 2 (TMPRSS2), in the pancreas of non-human primates (NHPs) and humans [23–26]. Of note, several of these studies showed that the entry factors were enriched in pancreatic microvascular and ductal structures, with little to no expression observed in endocrine cells [23, 24], while other studies demonstrated viral entry factor expression in endocrine cells [25, 26]. Together, these early studies led to uncertainty regarding the cellular target(s) of the SARS-CoV-2 virus within the pancreas.

Regardless of these inconsistencies, studies published in August 2021 provided evidence that SARS-CoV-2 could indeed infect human beta cells and alter cell function and pancreas health [27, 28]. Wu et al reported SARS-CoV-2-infected beta cells in individuals who succumbed to COVID-19 and showed that human islets could be infected ex vivo with SARS-CoV-2 [27]. Furthermore, this study showed that SARS-CoV-2 impacted beta cell function, as ex vivo infection of human islets resulted in increased beta cell death and decreased glucose-stimulated insulin secretion that was dependent upon the expression of the viral entry factor neurophilin-1 receptor, which was highly expressed in beta cells [27]. Additionally, a study by Tang and colleagues suggested that COVID-19 infection promoted beta cell transdifferentiation [28]. Using single-cell RNA sequencing, they showed that ex vivo infection of human islets with SARS-CoV-2 decreased beta cell expression of insulin, with a concomitant increase in markers of alpha cells (*GCG*) and acinar cells (*PRSS1*) [28].

As an alternative to direct effects on the beta cells, additional studies suggested that COVID-19 infection in the pancreas could alter vascular function, raising the possibility that damage to beta cells was the result of a ‘bystander effect’ [25, 29]. Pancreas tissues from NHPs and humans with COVID-19 showed viral infection in the islets, ductal tissue and endothelial cells, which resulted in fibrosis and vascular thrombi [25]. Interestingly, a significant portion of these SARS-CoV-2-infected NHPs and individuals developed new-onset diabetes [25]. A study published in 2025 by Andrade Barboza and colleagues further supported this link between COVID-19 infection and vascular dysfunction in the pancreas by showing that SARS-CoV-2 spike S1 recombinant protein activates pericytes in the pancreas, leading to islet capillary constriction and vascular dysfunction [29].

Taken together, these studies suggest that SARS-CoV-2 infection can directly perturb pancreatic function through multiple mechanisms, including viral infection of beta cells, alterations in beta cell identity, microvascular injury and impaired insulin secretion. Such perturbations could reduce functional beta cell mass or compromise insulin secretory capacity [20], findings that are present in both type 1 and type 2 diabetes. On the other hand, viral infections have also long been proposed as environmental triggers of autoimmune responses directed against pancreatic beta cells, while several viral pathogens have been implicated in the development of type 1 diabetes [30–32], raising the possibility that SARS-CoV-2 infection could influence glucose homeostasis through activation of immune-mediated beta cell destruction. However, at least one study did not support a robust response within pancreatic islets following SARS-CoV-2 infection. In single-cell transcriptomic and mass cytometry analyses of in vitro SARS-CoV-2-infected human islets, viral RNA was detected in multiple pancreatic cell types, yet relatively modest transcriptional and inflammatory responses were observed. These findings suggest that while SARS-CoV-2 can perturb beta cell function, the extent to which infection alone can initiate or sustain islet-directed immune responses in ex vivo studies remains uncertain [33].

### Effects of SARS-CoV-2 on systemic inflammation and peripheral insulin-sensitive tissues

Inflammatory and metabolic consequences of acute viral infection are not limited to the pancreas. Critical illness, regardless of aetiology, can trigger cytokine release and systemic inflammation that impairs insulin signalling, increases hepatic glucose production and reduces peripheral glucose uptake, resulting in insulin resistance and hyperglycaemia. In more severe illness, including systemic inflammatory response syndrome (SIRS) or sepsis, these effects are further amplified by activation of the hypothalamic–pituitary–adrenal (HPA) axis, leading to increased production of several counter-regulatory hormones, including glucocorticoids, catecholamines, growth hormone and glucagon, which exacerbate metabolic dysregulation [34]. Moreover, individuals hospitalised for COVID-19 often received pharmacological doses of glucocorticoids as part of their treatment; a known side effect of steroid treatment is hyperglycaemia [35]. As such, stress- and treatment-induced hyperglycaemia is common in critical illness, occurring in approximately 50% of affected individuals. While often transient, these effects may unmask persistent metabolic dysfunction in individuals at risk of type 2 diabetes [36, 37].

In addition to these systemic inflammatory responses, SARS-CoV-2 has been shown to directly infect peripheral insulin-sensitive tissues, including adipose tissue [38], skeletal muscle [39] and liver [40], thereby disrupting key pathways that regulate insulin sensitivity and glucose metabolism. In autopsy studies, SARS-CoV-2 RNA was identified in multiple adipose depots including epicardial, visceral and subcutaneous fat, where the presence of viral RNA was associated with inflammatory mononuclear cell infiltrate. In this same report, adipocytes and adipose-tissue macrophages were shown to be permissive to SARS-CoV-2 infection, where infection drove inflammatory responses in both cell types [41]. In addition to altered insulin signalling, acute COVID infection is also associated with reduced serum levels of several beneficial adipokines such as adiponectin and adipsin, suggestive of a generalised adipocyte dysfunction in addition to alterations in insulin sensitivity [42].

Similarly, studies indicated that SARS-CoV-2 viral proteins were found in skeletal muscle, leading to an array of abnormalities, including severe acute myopathy [43]. The structural and functional components of skeletal muscle impacted by COVID-19 include mitochondrial abnormalities, myopathic inflammation, cytokine production and muscle fibre atrophy [39, 43]. Additionally, there have been reports of elevated liver enzymes [40], as well as liver failure in individuals with cirrhosis [44, 45], following COVID-19 infection. At the cellular level, COVID-19 infection was associated with endoplasmic reticulum swelling and hepatocyte apoptosis in the liver [40, 46], suggesting there could be additional effects on insulin signalling in the liver. Along these lines, numerous publications reported links between SARS-CoV-2, carbohydrate metabolism, hyperglycaemia and insulin resistance. Several case reports emerged during the pandemic showing severe hyperglycaemia and insulin resistance in individuals with COVID-19 [47], and additional studies suggested abnormalities in glycometabolic control and insulin resistance following infection [48, 49]. Interestingly, worsened insulin sensitivity was observed also in children with pre-existing type 1 diabetes who presented in ketoacidosis, highlighting overlap between features of type 1 and type 2 diabetes during COVID-19 [50]. However, not all studies support this connection between COVID-19 and long-term changes in glucose homeostasis and insulin sensitivity [51, 52].

Overall, these findings demonstrate that SARS-CoV-2 is capable of disrupting glucose homeostasis through both systemic inflammatory responses and direct effects on peripheral insulin-sensitive tissues, thereby providing a mechanistic framework for the development of insulin resistance during and following infection. Together with studies performed in the pancreas, these mechanisms suggest that SARS-CoV-2-associated dysglycaemia reflects the convergence of impaired insulin secretion and reduced insulin sensitivity, with the relative contribution of each pathway likely varying across individuals and clinical contexts.

### Behavioural and environmental changes during the COVID-19 pandemic

In addition to these biological effects, societal, behavioural and psychosocial changes were prevalent during the COVID-19 pandemic. Public health measures implemented to limit viral transmission, including lockdowns, school closures, remote work and reduced access to outside activities, were frequently associated with reduced physical activity, altered dietary patterns and weight gain, all factors known to influence metabolic health and type 1 and type 2 diabetes risk [53, 54]. For example, children with high genetic susceptibility to type 1 diabetes participating in the Global Platform for the Prevention of Autoimmune Diabetes (GPPAD) Primary Oral Insulin Trial (POInT) exhibited increases in body mass index (BMI) during the pandemic period. Notably, higher BMI was associated with an increased risk of developing islet autoimmunity, suggesting that pandemic-related changes in weight and activity could influence pathways involved in the early stages of type 1 diabetes development.

Prior to the pandemic, type 2 diabetes incidence was noted to be declining in many populations; however, it is likely that pandemic shutdown-related weight gain, changes in healthcare access and disruptions in physical activity and eating patterns attenuated these decreased diabetes rates [53, 55, 56]. One study from Italy reported more than a doubling in type 2 diabetes incidence in the years following the COVID-19 pandemic compared with the proceeding years (incidence rate of 4.85 per 1000 person-years in 2017–2019 vs 12.21 per 1000 person-years in 2020–2022) [56]. Consistent with these observations, a retrospective analysis of youth from 23 clinical centres in the United States found that the frequency of both type 1 and type 2 diabetes increased during the pandemic. However, after accounting for pre-existing yearly trends, the increase was statistically significant only for type 2 diabetes. Notably, among youth with type 2 diabetes, BMI at presentation was higher during the pandemic period, supporting the possibility that pandemic-associated changes in lifestyle contributed to the increased incidence of type 2 diabetes [57, 58].

Finally, psychosocial stress during the pandemic has been associated with changes in neurobiological function and levels of HPA axis activation that could theoretically alter glucose homeostasis. These effects may be particularly relevant in children and adolescents, populations in which both psychosocial stress and behavioural changes during the pandemic were pronounced [57, 58]. Together, these observations suggest that the COVID-19 pandemic likely influenced type 1 and type 2 diabetes risk through a complex interaction of biological, behavioural, societal and psychosocial factors.

## Is there an epidemiologic association between SARS-CoV-2 infection and diabetes?

While mechanistic studies outline several plausible pathways through which SARS-CoV-2 could influence glycaemic management or diabetes development, they do not establish whether such effects occurred at the population level. Many epidemiologic studies have evaluated diabetes incidence following COVID-19 infection and have included studies from diverse global cohorts. These reports have largely used data based on retrospective analyses of clinical data, electronic health records, insurance claims data and registry data.

Table 1 summarises key meta-analyses evaluating the relationship between SARS-CoV-2 infection and diabetes, highlighting substantial heterogeneity in study design, case definitions, control groups, diabetes classification and outcome definitions that complicate interpretation of these findings. These meta-analyses support an association between SARS-CoV-2 infection and increased diabetes risk. However, the strength of this conclusion depends heavily on the quality of the underlying data. Many analyses were based on a small number of studies, with several reporting on overlapping studies. In addition, confirmation of COVID-19 status varied substantially across studies and meta-analyses, introducing the risk of misclassification bias, as undiagnosed or asymptomatic cases of COVID-19 may have been excluded. Look-back periods to determine whether diabetes was truly a new diagnosis were inconsistent, and methods to define diabetes and differentiate type 1 and type 2 diabetes varied widely across studies. A substantial proportion did not attempt to differentiate type 1 from type 2 cases.

**Table 1:** Summary of meta-analyses evaluating COVID-19 and new-onset diabetes

Author	Diabetes type	Year	Number of studies	Conclusions	Quality assessment	Assessment of publication bias
Rahmati et al [76]	T1D	2022	26	• Global incidence rate of T1D in 2019 was 19.73 per 100,000 children and 32.39 per 100,000 in 2020 • Authors reported results as a logit event rate of 0.080 (95% CI 0.028, 0.133)	No	Egger’s test *p*=0.443 overall
Rosenbauer et al [77]	T1D	2022	26	• Authors recalculated Rahmati 2022 [76] results in a letter to the editor and provided a revised incidence rate ratio of 1.119 (95% CI 1.046, 1.196)	No	No
D’Souza et al [78]	T1D	2023	17	• 38,149 cases of incident type 1 diabetes in youth • Incidence rate ratio of type 1 diabetes of 1.14 in the first year of the pandemic compared with pre-pandemic periods • Incidence rate ratio of 1.27 in months 13–24 of the COVID-19 pandemic • 26% increase in rates of diabetic ketoacidosis at diagnosis compared with pre-pandemic data	Yes, all studies assessed to have some bias	No
Rahmati [79]	T1D	2023	7	• Risk ratio of new‐onset type 1 diabetes following SARS‐CoV‐2 infection in children and adolescents was 1.42 (95% CI 1.13, 1.77) compared with non‐COVID‐19 control groups	NOS range 6–9 for individual studies	Egger’s test *p* non-significant
Zhang et al [80]	T1D and T2D	2022	9	• RR of diabetes after COVID-19 was 1.62 (95% CI 1.45, 1.80) compared with non-COVID-infected group • Type 1 diabetes RR 1.48 (1.26, 1.75) and type 2 diabetes RR 1.70 (1.32, 2.19)	Overall NOS of 88/90	Egger’s test *p*=0.104
Lai et al [81]	T1D and T2D	2023	10	• RR of 1.64 (95% CI 1.51, 1.79) for diabetes in those with COVID-19 compared with non-COVID-19-infected control individuals • Risk of type 2 diabetes > type 1 diabetes	Risk assessed using CLARITY group methods	Egger’s test *p*=0.054
Zhou et al [82]	T1D and T2D	2024	6	• HR for incident diabetes was 1.46 (95% CI 1.38, 1.55) • Type 1 diabetes HR 1.44 (95% CI 1.13, 1.82) • Type 2 diabetes HR 1.47 (95% CI 1.36, 1.59)	Average NOS of 8 for included studies	Egger’s test *p*=0.166
Cocking et al [83]	T1D and T2D	2025	12	• Risk ratio of 1.41 (95% CI 1.07, 1.84) • Subgroup analysis showed type 1 diabetes RR 0.96 (95% CI 0.45, 2.06) and type 2 diabetes was 1.31 (95% CI 1.07, 1.60) • Higher risk in adults and in those with higher severity of infection	Overall NOS 103/108	Egger’s test *p*=0.9766
El-Naas [84]	T1D and T2D	2025	35	• Overall prevalence of incident diabetes post COVID-19 was 1.37% • Sensitivity analysis revealed that corticosteroid use had no significant effect on diabetes incidence	NOS assessment showed wide range of quality (range 3–9)	No
Sathish et al [85]	Undifferentiated	2021	8	• 711 individuals with COVID‐19 and 492 cases of newly diagnosed diabetes; meta‐analysis estimated a pooled effect size of 14.4% (95% CI 5.9, 25.8)	Quality assessed as fair to good	No
Banerjee et al [86]	Undifferentiated	2022	4	• HR 1.59 (95% CI 1.40, 1.81) for development of diabetes compared with non-infected control population	NOS range 6–8	No
Ssentongo et al [87]	Undifferentiated	2022	8	• Risk ratio for incident diabetes 1.66 (95% CI 1.38, 2.00) in COVID-19 (4.27 million) vs non-COVID (43.2 million) infected youth and adults	Yes	Egger’s test *p*=0.053
Bellia et al [88]	Undifferentiated	2023	20	• The pooled proportion of new-onset diagnosis after at least 60 days from SARS-CoV-2 infection was 1.6% (95% CI 0.8, 2.7)	Only two studies were considered to be low risk of bias	No
Li et al [89]	Undifferentiated	2023	27	• RR of diabetes in COVID-infected vs non-infected control individuals was 1.75 (95% CI 1.43, 2.14)	Average AHRQ was 4.07 for cross-sectional studies Average NOS was 6.1 case–control and cohort	Egger’s test *p*=0.1971

AHRQ, Agency for Healthcare Research and Quality; T1D, type 1 diabetes; T2D, type 2 diabetes

In addition, control groups and comparison strategies were variable between the meta-analyses, and matching for key demographic parameters and exposures was similarly inconsistent. Some papers used historical incidence rates as comparators: an approach that can be problematic given that the incidence of both type 1 and type 2 diabetes fluctuates year-to-year. Other studies compared outcomes with a contemporaneous non-COVID-infected population. A more stringent but less common design would be to compare outcomes with individuals with another acute viral illness, as viral pathogens have long been implicated in the initiation of islet autoimmunity, type 1 diabetes and critical illness [30]. An example of this approach is a study by Lu et al that compared outcomes between 8216 hospitalised individuals, 2998 non-hospitalised individuals with COVID-19 and 2988 individuals hospitalised for influenza without a history of prediabetes or diabetes within the Montefiore Health System in New York. Persistent diabetes was diagnosed in 16.7% of hospitalised individuals with COVID-19 and 12% of hospitalised individuals with influenza. Notably, the adjusted odds ratio for developing persistent diabetes was not significant between the viruses [59]. Finally, even though the narrative of increased risk of type 1 diabetes was readily embraced by the academic and lay communities, only a small percentage of papers actually pursued biochemical confirmation of autoimmunity with autoantibody measurement. On the positive side, most of the meta-analyses included some type of quality assessment, typically the Newcastle–Ottawa Scale (NOS). In addition, the majority of these analyses assessed publication bias. Of the papers that assessed publication bias using funnel plots and Egger’s statistic, none found a significant *p* value, suggesting there was not over-representation of papers showing a positive association between COVID and diabetes. Taken together, while epidemiological data suggest a connection between COVID-19 and new-onset diabetes, there are numerous methodologic concerns that necessitate continued caution with the interpretation of this literature.

## Insights from prospective cohorts and longitudinal studies

High-quality evidence from prospective, longitudinal cohorts provides arguably the most rigorous data for assessing risk of diabetes after COVID-19 infection. Early during the pandemic, several ongoing type 1 diabetes clinical trials were able to add additional data collection to assess the impact of COVID-19 infection within their cohorts. In the European POInT/GPPAD cohort, SARS-CoV-2 seroconversion was associated with a striking increase in the development of islet autoimmunity, with a 3.5-fold elevation overall and an even stronger effect when infection occurred before 18 months of age [60]. Similarly, within the Fr1da cohort of children from the general population, in those with multiple islet autoantibody positivity (median age 4.1 years; IQR 3.0–5.3), the rate of progression to stage 3 type 1 diabetes after COVID-19 infection was nearly doubled [61]. However, in contrast to data from the GPPAD and Fr1da cohorts, COVID-19 infection did not appear to increase the risk of autoimmunity or diabetes development in adolescents from the TEDDY (The Environmental Determinants of Diabetes in the Young) cohort, which followed newborns from the USA and Europe with high genetic risk for type 1 diabetes [62]. These discordant findings in the TEDDY, GPPAD and Fr1da cohorts raise the possibility that any potential diabetogenic effect of COVID-19 may be age-dependent or confined to very early childhood.

Importantly, whether prevention of COVID-19 infection with vaccination in early life can modify the risk of islet autoimmunity in genetically susceptible infants is now being directly tested in a randomised controlled trial within GPPAD (AVAnT1A; ClinicalTrials.gov registration no. NCT06452654). In support of this approach, observational cohort studies suggest that COVID-19 vaccination prior to SARS-CoV-2 infection may reduce the risk of incident diabetes, with several large database analyses reporting lower rates of new-onset diabetes among vaccinated compared with unvaccinated individuals following infection [63–66]. Although rare cases of autoimmune diabetes have been reported following vaccination [67], overall, the literature has supported a substantial reduction in post-viral metabolic dysfunction in vaccinated individuals. However, a continued caution is that these data are derived primarily from retrospective or registry-based studies, and prospective evidence specifically designed to evaluate diabetes outcomes remains limited.

A complicating factor to many epidemiologic studies has been the changing identity and virulence of SARS-CoV-2 throughout the pandemic. The National COVID Cohort Collaborative (N3C) is a large, harmonised US research platform that aggregates electronic health record data from dozens of health systems to enable high-resolution analyses of COVID-19 outcomes and sequelae, including incident diabetes. Using data from the N3C platform, Wong et al evaluated the impact of SARS-CoV-2 infection on incident diabetes across different viral variant periods. Adults with confirmed COVID-19 were compared with COVID-negative control individuals as well as individuals with non-COVID acute respiratory illnesses, with follow-up extending to 1 year. Across all variants, SARS-CoV-2 infection was associated with a modest but statistically significant increase in the risk of new-onset diabetes (aggregating cases of type 1 and type 2 diabetes), with cumulative incidence ratios ranging from approximately 1.13 to 1.18. Although the overall magnitude of excess risk remained similar across variant eras, the timing of diabetes onset differed: earlier variants showed risk elevation within the first months after infection, whereas the Omicron variant period demonstrated a delayed increased risk, with most excess cases emerging approximately 180 days post COVID-19 infection [68]. To continue to study these complex relationships, other centres are attempting to aggregate data for long-term follow-up. An international group of diabetes researchers has established the CoviDIAB Project, which is a global registry of individuals with COVID-19-related diabetes (covidiab.e-dendrite.com). The goal of the registry is to describe the prevalence and characteristics of diabetes occurring after COVID-19 infection [69].

Finally, an additional area of interest in long-term follow-up studies is the potential relationship between long COVID and diabetes. Long COVID, or post-COVID-19 syndrome (PCS), occurs in an estimated 4–20% of infected individuals and is defined by new or persistent symptoms, including fatigue, chronic pain, memory and concentration issues, and respiratory symptoms, after a COVID-19 infection [70, 71]. Individuals with older age, female sex, elevated BMI and pre-existing conditions such as hypertension and diabetes are at higher risk for developing long COVID [72, 73]. Several mechanisms have been proposed to underlie long COVID pathogenesis, including vascular dysfunction, chronic low-grade inflammation and immune activation, autoantibody formation, nutrient deficiency and sustained dysregulation of tissue ACE2. Interestingly, many of these mechanisms overlap with those involved in diabetes pathogenesis and altered glucose homeostasis. Emerging data also raise the possibility that viral persistence within specific tissue compartments may contribute to ongoing metabolic dysfunction. Along these lines, adipose tissue has been proposed as a potential reservoir for viral components. Such persistence could provide a mechanistic link between prior infection and obesity [74]. While these observations suggest a similar bidirectional relationship between diabetes and long COVID [74], current evidence remains limited and largely observational. Future studies incorporating longitudinal metabolic phenotyping and mechanistic investigation will be required to determine whether long COVID represents a distinct pathway contributing to incident diabetes or reflects persistence of underlying metabolic vulnerability unmasked during acute infection.

## Conclusion and remaining challenges

Pelle and colleagues aptly described the intersection of the global COVID-19 pandemic and the longstanding diabetes epidemic as the collision of ‘two giants’ [75]. From early observations identifying diabetes as a major determinant of COVID-19 severity and mortality to subsequent reports linking SARS-CoV-2 infection with dysglycaemia and incident diabetes, this relationship has required rapid interpretation of evolving and sometimes conflicting data. Experimental studies provide biologically plausible mechanisms through which SARS-CoV-2 could disrupt glucose homeostasis, including effects on pancreatic, vascular and peripheral insulin-sensitive tissues. Effects that are indirect could also be important and include impacts on systemic inflammation, activation of the HPA and changes in lifestyle during the pandemic that impacted weight, food choice, stress and leisure activities. At the population level, many epidemiologic studies suggest an association between COVID-19 and new-onset type 1 and type 2 diabetes; however, substantial heterogeneity in study design, case definitions, comparator groups, inconsistent differentiation of type 1 and type 2 diabetes, and limited biochemical confirmation of autoimmunity constrain causal inference. Together, these findings indicate that SARS-CoV-2 is unlikely to represent a universal trigger for diabetes and that its effects on glycaemic management are unlikely to operate through a single pathway. Continued long-term follow-up, rigorous phenotyping and integration of mechanistic and epidemiologic data will be essential to define the true metabolic legacy of the COVID-19 pandemic. However, should future global pandemics arise, the lessons learned during the SARS-CoV-2 pandemic should hopefully serve as a blueprint for more robust, higher-quality surveillance of diabetes risk and disease development.

## Supplementary Information

Below is the link to the electronic supplementary material.

Figure slide (PPTX 196 KB)

## Abbreviations

ACE2: Angiotensin-converting enzyme 2
BMI: Body mass index
GPPAD: Global Platform for the Prevention of Autoimmune Diabetes
HPA: Hypothalamic–pituitary–adrenal
N3C: National COVID Cohort Collaborative
NHP: Non-human primate
NOS: Newcastle–Ottawa Scale
POInT: Primary Oral Insulin Trial
SARS-CoV-2: Severe acute respiratory syndrome coronavirus 2
TEDDY: The Environmental Determinants of Diabetes in the Young

## Authors’ relationships and activities

CE-M has served on advisory boards related to type 1 diabetes research clinical trial initiatives: Isla Technologies, DiogenyX, Neurodon, Sanofi; has received in-kind research support from BMS (access to compounds for a BT1D-funded project); and received past Investigator-initiated grants: Lilly Pharmaceuticals and Astellas Pharmaceuticals. CE-M has a patent (16/291,668) for Extracellular Vesicle Ribonucleic Acid (RNA) Cargo as a Biomarker of Hyperglycaemia and Type 1 Diabetes, and CE-M has a provisional patent (63/285,765) for Biomarker for Type 1 Diabetes (PDIA1 as a biomarker of beta cell stress). These activities have not dealt directly with topics covered in this manuscript. The authors declare that there are no other relationships or activities that might bias, or be perceived to bias, their work.
