# Supplementary material for: SARS-CoV-2 and diabetes: a post-pandemic reappraisal
Source: Diabetologia. 2026 Jun 27;69(9):2396–407. doi: 10.1007/s00125-026-06785-4 (PMC13424210; doi:10.1007/s00125-026-06785-4)
Supplement: Supplementary file 1 — Figure slide (PPTX 196 KB) [file 125_2026_6785_MOESM1_ESM.pptx]

## Slide 1
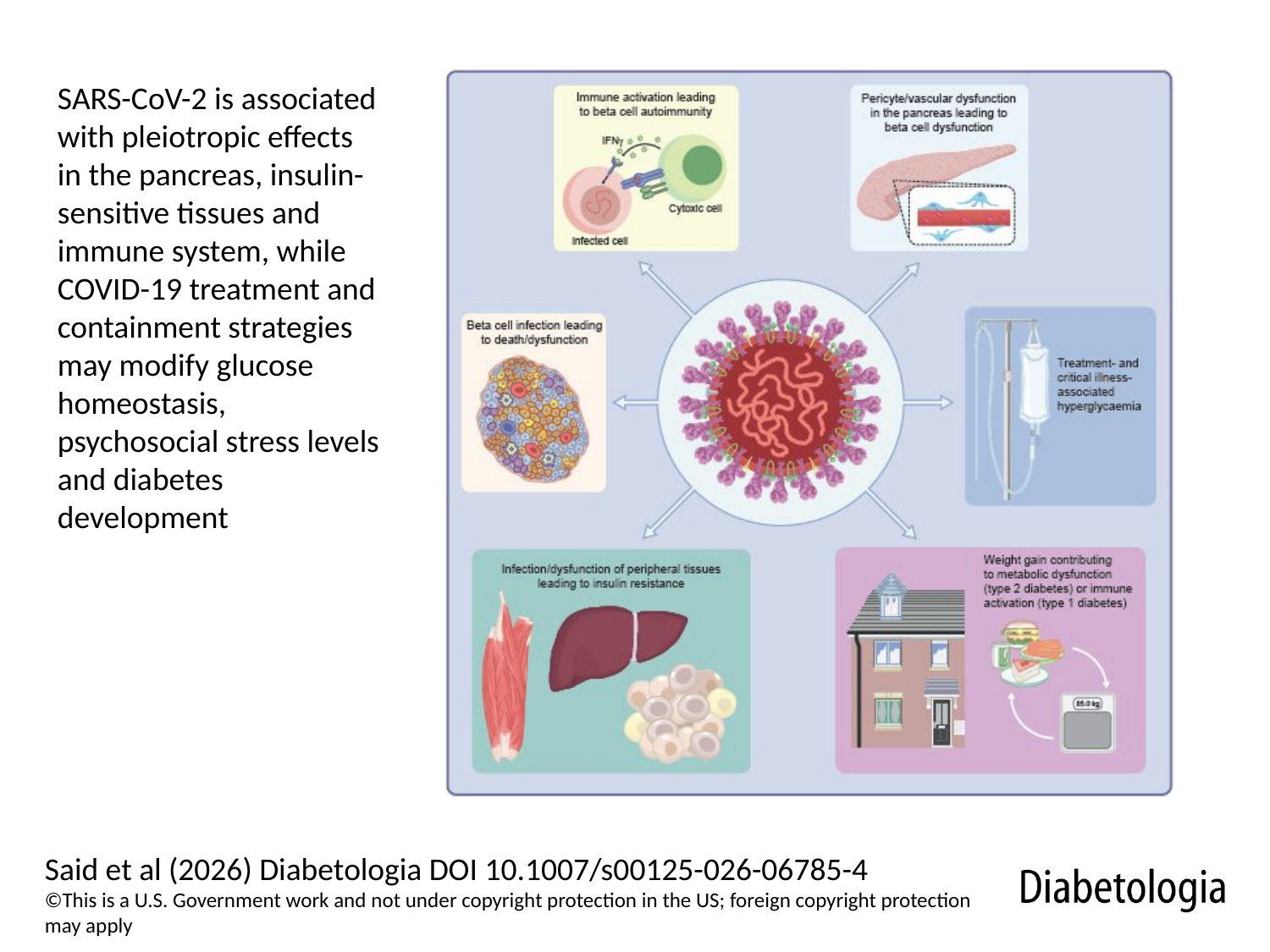

SARS-CoV-2 is associated with pleiotropic effects in the pancreas, insulin-sensitive tissues and immune system, while COVID-19 treatment and containment strategies may modify glucose homeostasis, psychosocial stress levels and diabetes development
Said et al (2026) Diabetologia DOI 10.1007/s00125-026-06785-4
©This is a U.S. Government work and not under copyright protection in the US; foreign copyright protection may apply
